# The Active Classroom: A Narrative Review of Active Teaching Methods and the Flipped Classroom Model in Graduate Medical Education

**DOI:** 10.7759/cureus.98186

**Published:** 2025-11-30

**Authors:** Kalyan Kandra, Praneetha Vennam

**Affiliations:** 1 Psychiatry, Southern Illinois University School of Medicine, Springfield, USA; 2 Center for Clinical Research, Southern Illinois University School of Medicine, Springfield, USA

**Keywords:** active learning methods, active teaching methods, case-based learning, flipped classrom, graduate medical education, problem-based learning (pbl), team-based learning (tbl)

## Abstract

Graduate medical education (GME) is undergoing a significant pedagogical transformation, moving away from traditional, passive learning environments toward more dynamic, learner-centered approaches. This narrative review examines the implementation and impact of active teaching methods in GME, with a specific focus on the flipped classroom model. In this narrative review, we compare and contrast these innovative strategies with traditional didactic lectures, evaluating their effects on learner engagement, knowledge retention, clinical reasoning, and overall satisfaction. Active learning, grounded in constructivist theory, repositions the resident as an active participant in their education, utilizing methods such as case-based learning, team-based learning, and simulation. The flipped classroom, a prominent example, inverts the conventional educational structure by delivering foundational content outside the classroom to reserve in-person time for collaborative problem-solving and application of knowledge. While traditional methods are efficient for information dissemination, evidence suggests that active learning models foster the development of higher-order thinking and essential clinical competencies. Successfully overcoming implementation challenges requires a strategic approach centered on faculty and learner engagement, continuous feedback, and robust institutional support. This review concludes that the adoption of active teaching methodologies is critical for preparing future physicians with the skills necessary to navigate the complexities of modern healthcare.

## Introduction and background

For generations, the bedrock of medical education has been the traditional didactic lecture, a model where knowledge is transmitted from an expert instructor to a passive audience of learners. This “sage on the stage” approach, while effective for conveying large volumes of information efficiently, often promotes rote memorization over deep conceptual understanding and critical inquiry [1]. Its persistence is understandable; it is a familiar, scalable, and structured method for delivering a standardized curriculum, tracing its roots to a time when information was scarce and the expert was the primary conduit. However, in the context of graduate medical education (GME), where the goal is to cultivate competent, autonomous physicians, there is a growing consensus that this passive model is insufficient. It struggles to develop the complex clinical reasoning, collaborative skills, and lifelong learning habits required in contemporary medical practice [2,3]. The modern clinical environment is not a passive lecture; it is a dynamic, collaborative, and often ambiguous setting that demands more than just recall of facts. Passive learning often fails to build cognitive flexibility, leaving residents well-versed in textbook facts but ill-equipped to apply that knowledge to novel, complex patient presentations that do not follow a script.

In response, a paradigm shift is occurring, favoring the adoption of active learning strategies. This shift is grounded in constructivist learning theory, which posits that learners build new knowledge upon their existing framework of understanding, not by passively absorbing information but by actively making sense of it [4]. Active learning is a broad term for instructional methods that involve students in the learning process directly. Instead of just listening, students are engaged in activities such as reading, writing, discussion, or problem-solving that promote analysis, synthesis, and evaluation of class content [5]. This pedagogical approach reframes the instructor’s role from a dispenser of information to a “guide on the side,” facilitating a more interactive and collaborative learning environment. This shift is driven not only by evolving educational theory but also by institutional and accreditation standards. These standards, which explicitly include skills like practice-based learning, interpersonal communication, and systems-based practice, are difficult to foster in a purely didactic setting. A lecture, for instance, cannot effectively teach a resident how to communicate difficult news or collaborate with an interprofessional team; these are skills that must be practiced, observed, and refined through active participation. This review will explore the spectrum of active teaching methods in GME, provide an in-depth analysis of the flipped classroom model, and contrast these approaches with traditional teaching methods.

## Review

The spectrum of active learning in graduate medical education

The fundamental difference between traditional and active learning lies in the role of the learner. In the traditional lecture hall, the resident is often a passive recipient, with learning measured by the ability to recall information. In contrast, active learning requires the resident to be an active participant, constructing their own knowledge through direct engagement with the material and collaboration with peers [5]. This approach encompasses a wide spectrum of methods, from simple in-class techniques to complex curricular structures.

Key Examples of Graduate Medical Education

Problem-based learning (PBL): Residents are presented with an ill-structured clinical case before any formal instruction. Working in small groups, they must analyze the case (which may “unfold” with new information over time), identify knowledge gaps (e.g., “What do we need to know about the pathophysiology of this symptom?”), and define their own learning objectives. The group then engages in self-directed learning to research these objectives before reconvening to apply their new knowledge to solve the case. The facilitator’s role is not to provide answers but to guide the inquiry process with probing, Socratic questions (e.g., “What is the evidence for that?” or “What alternatives have you considered?”), thereby mirroring the “diagnostic reasoning” required in clinical practice [6].

Team-based learning (TBL): A highly structured, multi-phase method. Before class, learners complete pre-work. In class, the process begins with an individual readiness assurance test (iRAT), a short quiz on the pre-work. Immediately after, learners take the same test as a team (tRAT), coming to a consensus on each answer. This process provides immediate, corrective feedback and fosters robust peer teaching. The bulk of the session is then spent with these same teams working on complex application exercises (e.g., analyzing a challenging case, comparing treatment protocols) where they must make a specific choice and defend it. This method promotes individual accountability (via the iRAT) and sophisticated collaborative problem-solving [7].

Case-based learning (CBL): Often used as a bridge between didactic learning and pure PBL. In CBL, foundational knowledge (e.g., a lecture or reading on a specific disease) is typically provided beforehand. Residents then work through guided clinical cases, often with specific questions, to apply this new knowledge. CBL is generally more directed than PBL and is highly effective for reinforcing key teaching points and demonstrating how foundational theory translates to its direct clinical application.

Simulation-based learning: This method provides an immersive, hands-on experience in a safe, controlled environment, allowing for “deliberate practice” without patient risk. Simulations can range from low-fidelity task trainers (e.g., for suturing) to high-fidelity mannequins that mimic physiological responses (e.g., for a code blue or an anesthesia crisis) to standardized patients (actors trained to portray specific clinical scenarios) for practicing communication skills (e.g., breaking bad news). The most critical component is the structured, post-simulation debriefing, where participants reflect on their actions, thought processes, and teamwork in a psychologically safe setting, solidifying the learning and identifying areas for improvement [8].

Think-pair-share: A simple yet powerful technique to break up a lecture and generate engagement. The instructor poses a complex, open-ended question. Learners are given a moment to think individually (e.g., “What are the top three differential diagnoses?”). They then pair with a partner to discuss their thoughts, refining their ideas. Finally, pairs share their conclusions with the larger group. This simple structure encourages universal participation (as opposed to just the “gunners”), lowers the anxiety of speaking in front of a large group, and allows learners to refine their understanding through peer dialogue before a public declaration [5].

Jigsaw: A collaborative method that builds both expertise and interdependence. A complex topic (e.g., different classes of antihypertensives) is divided into smaller pieces. Learners are first organized into “home groups.” They then break apart and re-form into “expert groups,” each of which is assigned one piece of the topic to master. Once they have become “experts,” they return to their “home groups” to teach their piece to their group-mates. This method makes each learner responsible for a component of the material, turning them into a teacher and promoting a deeper level of understanding [4,5].

Peer instruction: This method typically involves the instructor posing a challenging conceptual multiple-choice question. Learners first vote individually. Then, without revealing the correct answer, the instructor has the learners discuss their reasoning with their neighbors, particularly those who chose a different answer. After a few minutes of discussion, the instructor re-polls the class. This “peer-to-peer persuasion” is often highly effective at correcting misconceptions and forces learners to articulate and defend their reasoning [4].

The flipped classroom: a detailed examination of its advantages and usefulness

Among these various strategies, the flipped classroom model has gained significant traction for its transformative potential [3,9]. It is not a single teaching method but rather a structural model that inverts the traditional educational flow. By assigning foundational learning (e.g., recorded lectures, readings, or interactive modules) as pre-work, it reclaims valuable in-person time for targeted, high-impact activities that draw upon the other active methods mentioned above (e.g., using in-class time for case-based learning, TBL application exercises, or mini-simulations). The model’s usefulness stems from a range of interconnected advantages.

Deepens Learning and Enhances Knowledge Retention

The flipped classroom moves beyond surface-level memorization and aligns with higher-order cognitive processes, as described in Bloom’s Taxonomy. The pre-class work handles the lower-level tasks of “Remembering” and “Understanding.” The in-class session, now freed from information delivery, is dedicated to “Applying,” “Analyzing,” “Evaluating,” and “Creating.” This educational process is split into two crucial phases that align with how the brain learns best. Phase one, the pre-class work, provides initial exposure to concepts [10]. Phase two, the in-class session, requires residents to actively retrieve that information to solve problems, analyze cases, or defend a clinical decision. This act of retrieval practice is a powerful tool for cementing knowledge in long-term memory, fighting the natural tendency of the “forgetting curve” that plagues passively received information [11]. This process directly mimics the real-world clinical scenario where a physician must retrieve and apply knowledge under pressure, rather than simply recognizing it on a multiple-choice test.

Develops Critical Clinical Reasoning Skills

Medicine requires the ability to navigate ambiguity, synthesize disparate data, and generate differential diagnoses. The flipped classroom’s active session is a perfect laboratory for these skills. In-class time can be spent on complex, “gray area” cases, differential diagnosis debates, treatment plan formulations, or discussions of clinical dilemmas. The instructor, freed from lecturing, can act as an expert guide, posing Socratic questions to probe thinking (“What evidence supports that diagnosis over the others?”), correcting flawed logic in real-time, and modeling the sophisticated thought processes of an experienced clinician. This is a form of cognitive apprenticeship, making the expert’s implicit thought processes explicit for the novice, which is nearly impossible to replicate in a standard lecture.

Promotes a Collaborative Learning Culture

Modern medicine is a team sport, yet traditional lectures are often an individual, isolating experience. The flipped classroom is inherently collaborative. The in-class group work necessitates communication, teamwork, and the respectful debate of differing viewpoints. Residents learn from the diverse experiences and knowledge bases of their peers, building a “community of practice” that mirrors the functioning of a high-performing clinical team. This directly supports the development of interprofessional competencies, as residents learn how to learn from and with each other, consult colleagues, and reach evidence-based consensus.

Offers Personalized and Self-Directed Learning

The one-size-fits-all pace of a traditional lecture is a significant limitation, boring some learners while leaving others behind. The flipped classroom introduces a crucial element of personalization. During the pre-class work, residents can learn at their own pace, pausing, rewinding, and re-watching material as needed to ensure they have a solid grasp of the basics before the group session. This fosters learner autonomy and self-directed learning, a critical skill for lifelong learning in an ever-evolving medical field [10,12]. This personalization also benefits faculty, who can often use pre-class quizzes to identify common misconceptions before the session, allowing them to tailor the in-class activities to address those specific knowledge gaps.

Maximizes the Value of Expert Faculty Time

An expert clinician’s most valuable asset is their accumulated wisdom and clinical experience. The flipped model makes far more efficient use of this precious resource. By offloading the basic information transfer to pre-class materials (which can be recorded once and reused), faculty can dedicate their in-person time to the activities that require their true expertise: mentoring, facilitating complex discussions, managing “gray area” cases, and sharing nuanced “clinical pearls” that are not found in textbooks [2]. This can also lead to higher faculty satisfaction, as many educators find these interactive, facilitative sessions more engaging and rewarding than delivering the same lecture repeatedly.

Overcoming challenges: a roadmap for effective implementation

Despite the clear benefits, transitioning to active learning models presents challenges. These include the significant initial investment of faculty time to create high-quality, engaging pre-work, the need for new assessment methods that align with higher-order thinking, and potential resistance from both faculty and learners accustomed to traditional pedagogy. For residents, pre-work can feel like “more work” if not designed efficiently, and some may experience anxiety or discomfort with the ambiguity of inquiry-based methods, preferring the comfort of passive learning. For faculty, resistance can stem from a lack of technical skills, a fear of “losing control” of the classroom, or institutional promotion criteria that may not adequately reward the effort of educational innovation.

However, these obstacles are surmountable with a deliberate and supportive implementation strategy. Success hinges on a continuous cycle of engagement, feedback, and resource allocation.

The first step is to foster a culture of partnership through proactive engagement with both faculty and learners. For faculty, this involves practical workshops on instructional design, peer mentoring from early adopters, and a faculty learning community to share successes and troubleshoot failures. This support helps them transition from lecturer to facilitator. For residents, transparent communication about the model’s rationale (“why are we doing this?”) and clear expectations are crucial to secure their buy-in and ensure they are prepared for a more active role. Involving residents in the co-creation of modules can be particularly effective. This collaborative dialogue allows for adjustment of the learning environment and addresses concerns about workload and new responsibilities from the outset.

Crucially, implementation must be an iterative process guided by regular feedback. Rather than waiting for end-of-course evaluations, programs should create channels for continuous, real-time feedback through short anonymous surveys (“Was the pre-work too long/too short?”), mid-module focus groups, or informal check-ins. This data is invaluable for refining pre-class materials, adjusting the difficulty of in-class activities, and ensuring the model remains responsive to the evolving needs of the learners.

Finally, engagement and feedback must be backed by tangible support and resources from the institution. This includes providing faculty with protected time for curriculum development, a critical factor often overlooked in the calculus of implementation. Furthermore, institutions must provide robust technological and administrative support, such as access to video creation software, assistance from instructional designers, and a reliable learning management system. A key, and often forgotten, component is the alignment of assessment. If teaching methods focus on higher-order thinking, but exams only test for factual recall, the initiative will be undermined. This requires moving beyond simple multiple-choice questions to include assessments such as Objective Structured Clinical Examinations, team-based project evaluations, or portfolios of applied work. By investing in the people (through training and time), the infrastructure (through tools), and the assessment (through alignment), an institution signals its commitment to modern pedagogy and empowers its educators to implement these highly effective teaching methods successfully.

## Conclusions

The movement toward active learning in GME represents a necessary and fundamental evolution in medical pedagogy, moving from a 20th-century model of information transfer to a 21st-century model of knowledge construction and application. Models such as the flipped classroom, supported by a spectrum of methods from TBL to simulation, reframe education from a passive transfer of information to an active process of inquiry, application, and collaboration. While traditional lectures may still be useful for certain purposes (e.g., a “grand rounds” presentation of new research), active learning strategies are demonstrably superior for developing the essential, durable skills of clinical reasoning, critical thinking, problem-solving, and teamwork. The transition is not without its hurdles, requiring a significant cultural, logistical, and institutional commitment. However, the demands of modern healthcare, with its complex patients, team-based environments, and explosion of new information, require physicians who are adaptive, self-directed, and collaborative. By embracing these methods, strategically navigating the implementation process with strong institutional support, and committing to a culture of continuous feedback and improvement, GME programs can more effectively prepare residents for the complexities and demands of modern medical practice. This fosters a generation of physicians equipped not just with knowledge but with the wisdom and skills to apply it thoughtfully, collaboratively, and effectively throughout their careers.

## References

[1] Prober CG, Heath C (2012). Lecture halls without lectures--a proposal for medical education. N Engl J Med.

[2] Chen F, Lui AM, Martinelli SM (2017). A systematic review of the effectiveness of flipped classrooms in medical education. Med Educ.

[3] Tolks D, Schäfer C, Raupach T (2016). An introduction to the inverted/flipped classroom model in education and advanced training in medicine and in the healthcare professions. GMS J Med Educ.

[4] Freeman S, Eddy SL, McDonough M, Smith MK, Okoroafor N, Jordt H, Wenderoth MP (2014). Active learning increases student performance in science, engineering, and mathematics. Proc Natl Acad Sci U S A.

[5] Bonwell CC, Eison JA (1991). Active Learning: Creating Excitement in the Classroom. ASHE-ERIC Higher Education Report No. 1. Proc Natl Acad Sci U S A.

[6] Wood DF (2003). Problem based learning. BMJ.

[7] Parmelee DX, Michaelsen LK (2010). Twelve tips for doing effective team-based learning (TBL). Med Teach.

[8] Issenberg SB, McGaghie WC, Petrusa ER, Lee Gordon D, Scalese RJ (2005). Features and uses of high-fidelity medical simulations that lead to effective learning: a BEME systematic review. Med Teach.

[9] O'Flaherty J, Phillips C (2015). The use of flipped classrooms in higher education: a scoping review. Internet High Educ.

[10] Ramnanan CJ, Pound LD (2017). Advances in medical education and practice: student perceptions of the flipped classroom. Adv Med Educ Pract.

[11] Hew KF, Lo CK (2018). Flipped classroom improves student learning in health professions education: a meta-analysis. BMC Med Educ.

[12] Rotellar C, Cain J (2016). Research, perspectives, and recommendations on implementing the flipped classroom. Am J Pharm Educ.

